# Severe congenital *RYR1*-associated myopathy complicated with atrial tachycardia and sinus node dysfunction: a case report

**DOI:** 10.1186/s13052-019-0756-1

**Published:** 2019-12-19

**Authors:** Itaru Hayakawa, Yuichi Abe, Hiroshi Ono, Masaya Kubota

**Affiliations:** 1 Division of Neurology, National Center for Child Health and Development, 2-10-1 Okura, Setagaya-ku, Tokyo, 157-8535 Japan; 2 Division of Cardiology, National Center for Child Health and Development, 2-10-1 Okura, Setagaya-ku, Tokyo, 157-8535 Japan

**Keywords:** RYR1, Sinus node dysfunction, Atrial tachycardia, Congenital myopathy

## Abstract

**Background:**

Cardiac arrhythmias are sometimes encountered in patients with hereditary myopathies and muscular dystrophies. Description of arrhythmias in myopathies and muscular dystrophies is very important, because arrhythmias have a strong impact on the outcomes for these patients and are potentially treatable.

**Case presentation:**

A girl with severe congenital *RYR1*-related myopathy exhibited atrial tachycardia and sinus node dysfunction during infancy. She was born after uncomplicated caesarian delivery. She showed no breathing, complete ophthalmoplegia, complete bulbar paralysis, complete facial muscle paralysis, and extreme floppiness. At 5 months old, she developed persistent tachycardia around 200–210 beats per minutes. Holter monitoring revealed ectopic atrial tachycardia during tachyarrhythmia and occasional sinus pauses with junctional escape beats. Propranolol effectively alleviated tachyarrhythmia but was discontinued due to increased frequency and duration of the sinus pauses that led to bradyarrhythmia. There was no evidence of structural heart diseases or heart failure. The arrhythmia gradually resolved spontaneously and at 11 months old, she showed complete sinus rhythm.

**Conclusions:**

Although supraventricular arrhythmia is sometimes encountered in congenital myopathies, this is the first report of cardiac arrhythmia requiring drug intervention in *RYR1*-associated myopathy.

## Introduction

Cardiac arrhythmias are sometimes encountered in patients with hereditary myopathies and muscular dystrophies. Because of the increasing lifespan of these patients, prevalence of cardiac involvement is also increasing. Because arrhythmias have a strong impact on the outcomes for these patients and are potentially treatable, description of arrhythmias in myopathies and muscular dystrophies is very important [1, 2]. To date, arrhythmia has never been reported in cases of congenital *RYR1*-related myopathy.

Here we report a girl with severe congenital *RYR1*-related myopathy [2–4] who exhibited atrial tachycardia and sinus node dysfunction without cardiomyopathy during infancy.

## Case presentation

**Fig. 1:**
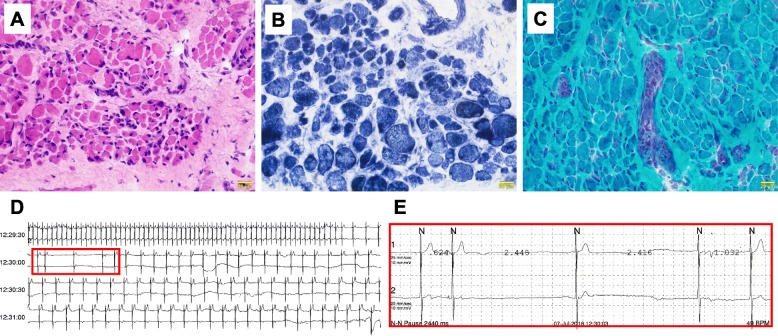
**a**–**c** Skeletal muscle (thyrohyoid muscle) histopathology was consistent with centronuclear myopathy. **d** and **e** Holter monitoring demonstrates sinus pause and junctional rhythms, confirming the diagnosis of sinus node dysfunction. **a**, hematoxylin & eosin; **b**, NADH-TR; **c**, modified Gomori-trichrome; calibration bar 20 μm; NADH-TR, nicotinamide adenine dinucleotide dehydrogenase - tetrazolium reductase.

The female patient was born to non-consanguineous healthy parents by caesarian delivery due to breech presentation at 36 weeks 3 days of gestational age. Polyhydramnios and decreased fetal movements were pointed out during pregnancy. After delivery, she showed neither spontaneous breathing nor spontaneous limb movements. She received standard resuscitative measures. On examination, no spontaneous breathing, complete ophthalmoplegia, complete bulbar paralysis, complete facial muscle paralysis, and extreme floppiness with diminished tendon reflexes were noted. Height, weight, and head circumference were within normal limits. No dysmorphic features were present. Severe congenital myopathy was suspected. She underwent muscle biopsy and genetic analysis that resulted in a diagnosis of severe congenital *RYR1*-associated centronuclear myopathy (Fig. 1a–c). She inherited compound heterozygous pathogenic or likely-pathogenic rare variants in the *RYR1* gene (*RYR1* NM_000540, p.D2529N from her father, p.L2155P and p.R682P in *cis* from her mother). The other genes in the congenital myopathy and myasthenia panel were negative.

At the age of 5 months old, she developed persistent tachycardia around 200–210 beats per minutes (bpm). She did not show fever, hypotension, abnormal urination, stool abnormality, diaphoresis, or mydriasis during the tachycardia. Routine laboratory investigation failed to show any extra-cardiac cause of tachycardia including infection, hyperthyroid status, or anemia. Holter monitoring revealed ectopic atrial tachycardia during tachyarrhythmia up to 200–210 bpm, junctional escape beats with sinus pauses after cessation of atrial tachycardia, and occasional sinus bradycardia around 50 bpm. Cardiac ultrasonography showed no evidence of structural heart disease or cardiomyopathy. The tachycardia was refractory to intravenous adenosine triphosphate trial and to intravenous procainamide. Atrial tachycardia and sinus node dysfunction, due presumably to her underlying severe congenital *RYR1*-associated centronuclear myopathy, were diagnosed.

Administration of propranolol (0.5–1.0 mg/kg/day) was initiated and was effective. Her heart rate lowered from 200-210 bpm to 130–150 bpm. Unfortunately, paroxysmal bradycardia around 30 bpm for several seconds began to occur almost daily after propranolol treatment. Repeated Holter monitoring revealed alleviation of atrial tachycardia, but increased frequency and duration of the sinus pauses with junctional rhythms that led to bradyarrhythmia (maximum R-R interval of 2.44 s) (Fig. 1d, e). As the patient had complete ophthalmoplegia and limb weakness, we could not determine whether the bradycardia was associated with any other signs or symptoms. Propranolol was discontinued at the age of 6 months. Fortunately, severe tachyarrhythmia did not recur after propranolol discontinuation. Her heart rates were mostly around 170–180 bpm, with occasional episodic drops down to 50–60 bpm. She was discharged home when she was 10 months old, with a tracheostomy, home respirator support, and a gastric tube. At planned follow-up (11 months old), Holter monitoring revealed complete sinus rhythm with occasional sinus bradycardia down to 50 bpm. She was still completely ophthalmoplegic, but her weakness improved slightly, and she and her family could communicate with each other by her subtle hand gestures and very slight facial muscle movements.

## Discussion and conclusions

This is to our knowledge the first report of arrhythmia requiring drug intervention in *RYR1*-associated myopathy [3, 4]. Although supraventricular arrhythmia is sometimes encountered in congenital myopathies [5], the reason why *RYR1* mutation led to atrial tachycardia and sinus node dysfunction in our patient remains unclear. *RYR1* is expressed predominantly in skeletal muscles and to a lesser extent in vascular smooth muscles, but does not exert any effects on cardiac muscles [6, 7].

Because arrhythmias have a strong impact on the outcomes for myopathy patients and are potentially treatable, early recognition and treatment of primary cardiac arrhythmia in myopathy patients is vitally important. We identified our patient’s atrial tachycardia with sinus node dysfunction through bedside observation and Holter monitoring. Prompt treatment with low-dose propranolol effectively alleviated the atrial tachycardia. The heart rate abnormality in congenital myopathy patients is usually a result of some respiratory or gastrointestinal infection. The otherwise unexplained tachycardia in severely affected myopathy patients may easily be overlooked to the point where tachycardia-induced heart failure becomes evident. We suggest that neurologists should be aware of the possibility of primary cardiac arrhythmias and perform Holter monitoring in severely affected congenital myopathy patients with an otherwise unexplained heart rate abnormality.

## Data Availability

All the data presented in this article is stored in our Unit.
